# Supplementary material for: TMIGD2 is an orchestrator and therapeutic target on human acute myeloid leukemia stem cells
Source: Nat Commun. 2024 Jan 2;15:11. doi: 10.1038/s41467-023-43843-6 (PMC10761673; doi:10.1038/s41467-023-43843-6)
Supplement: Supplementary file 1 — Supplementary Information file [file 41467_2023_43843_MOESM1_ESM.pdf]

Supplementary Information for

TMIGD2 is an orchestrator and therapeutic target on human acute myeloid leukemia stem cells

Hao Wang, R. Alejandro Sica, Gurbakhash Kaur, Phillip M. Galbo Jr, Zhixin Jing, Christopher D. Nishimura, Xiaoxin Ren, Ankit Tanwar, Bijan Etemad-Gilbertson, Britta Will, Deyou Zheng, David Fooksman, Xingxing Zang

Corresponding author: Xingxing Zang, xingxing.zang@einsteinmed.edu

This PDF file includes:

Supplementary Figures 1-7
Supplementary Tables 1-4

a

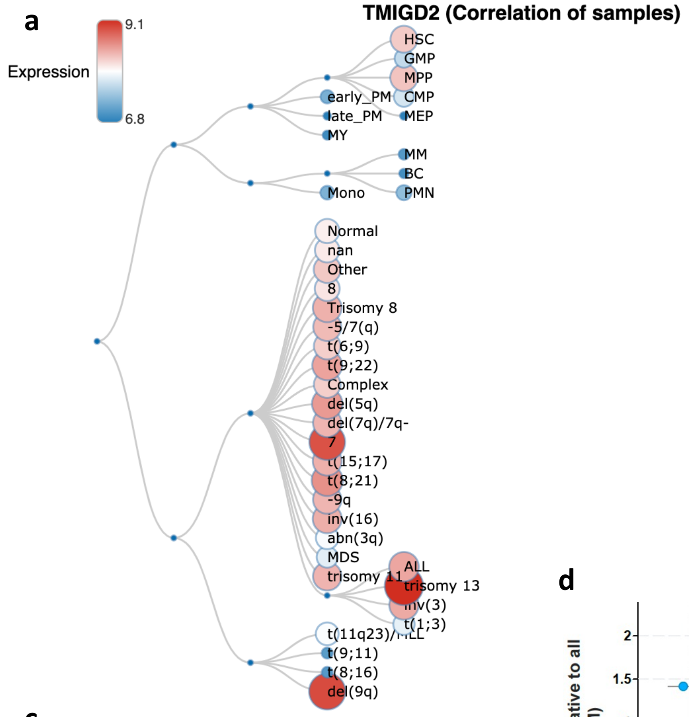

b

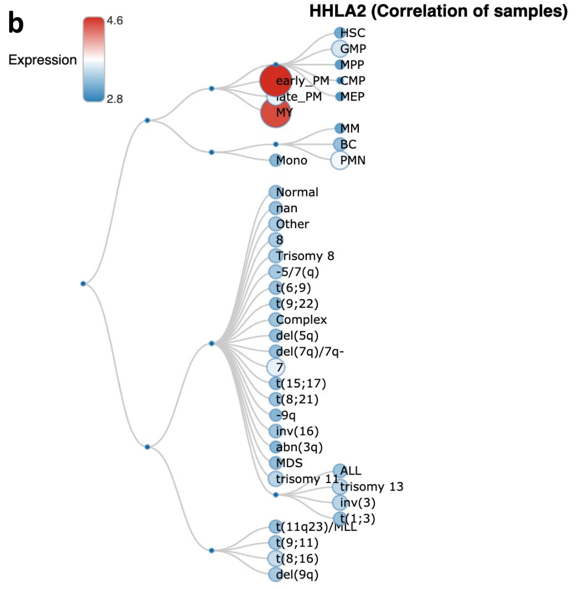

c

Gene Correlations

Value	Gene	Probe
0.4976	ITM2C	221004_s_at
0.483609	PTPRCAP	204960_at
0.471524	CBX2	240084_at
0.47023	FAM69B	229002_at
0.469182	SHANK3	227923_at
0.468628	CD34	209543_s_at
-0.466889	SNX10	218404_at
-0.469591	SUCLG2	212459_x_at
-0.469757	SUCLG2	215772_x_at
-0.479928	HNMT	204112_s_at
-0.491482	MPP7	1564308_a_at
-0.533667	MPP7	238778_at

d

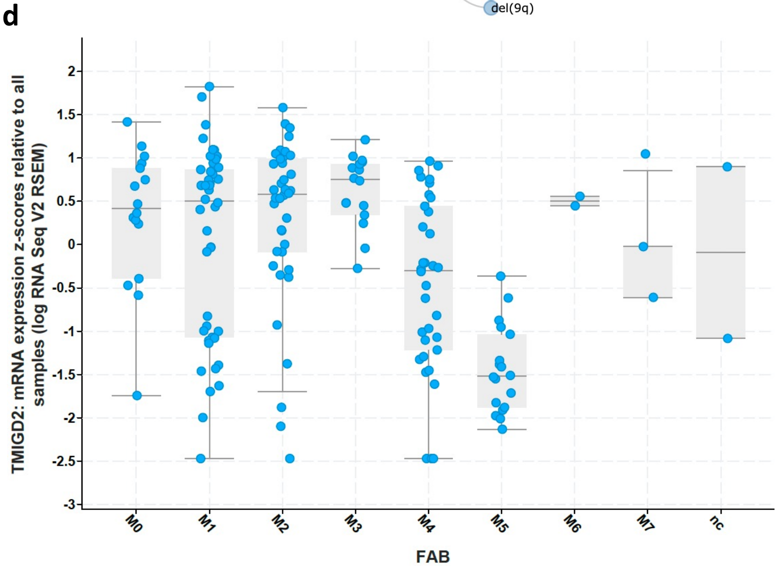

e

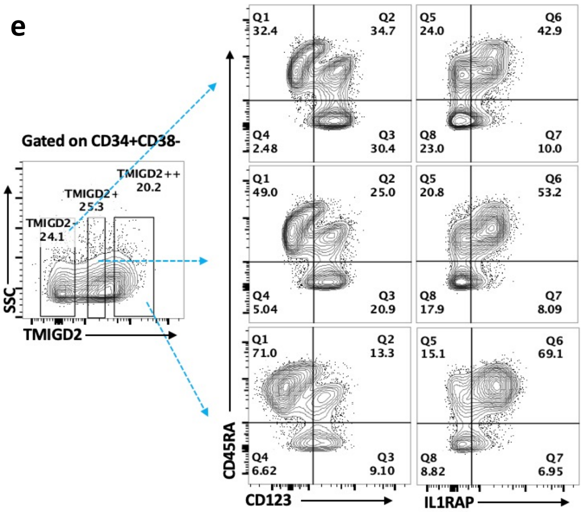

f

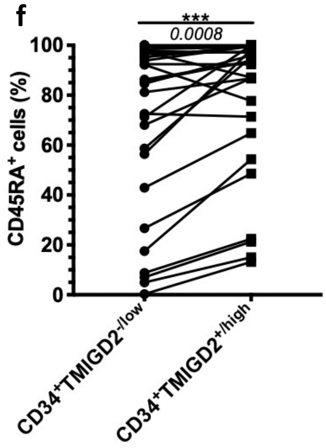

g

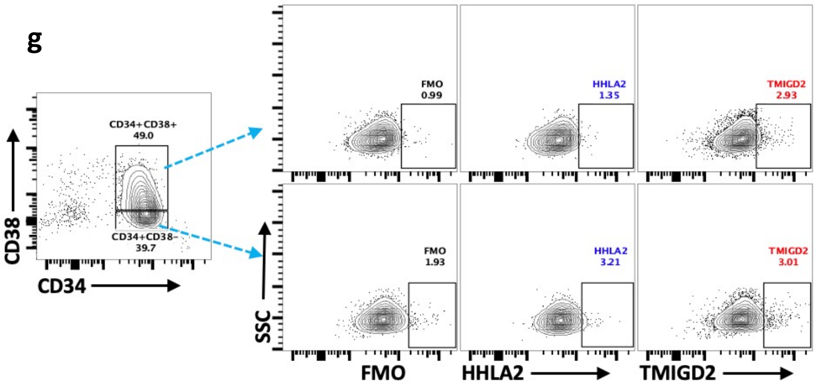

Supplementary Fig. 1 | The expression pattern of HHLA2 and TMIGD2 in human AML patients and normal stem cells.

a, Analysis of the BloodSpot database (www.bloodspot.eu) showing the increased TMIGD2 mRNA expression in primary human AML across cytogenetic subtypes in comparison with normal hematopoietic cells.

b, mRNA level of HHLA2 in normal hematopoietic and AML cells from BloodSpot database.

c, Gene correlation analysis of the BloodSpot database showing positive correlation between TMIGD2 and CD34.

d, mRNA expression of TMIGD2 in the AML samples/patients classified into AML subtypes (FAB classification). M0, n = 16; M1, n = 42; M2, n = 39; M3, n = 16; M4, n = 35; M5, n = 18; M6, n = 2; M7, n = 3; and two not-classified (nc) AML.

e, Representative flow cytometry gating strategy showing expression pattern of TMIGD2, CD45RA, CD123, and IL1RAP in CD34+CD38- subset from AML patient. TMIGD2-, TMIGD2 negative cells; TMIGD2+, TMIGD2 intermediate cells; TMIGD2++, TMIGD2 high cells.

f, CD45RA expression in $CD34^{+}TMIGD2^{-/low}$ versus $CD34^{+}TMIGD2^{+/high}$ cells from AML patients (n=31). ***$p < 0.001$ by paired Student's t test.

g, Representative flow cytometry gating strategy showing FMO control, HHLA2 and TMIGD2 expression in $CD34^{+}CD38^{-}$ and $CD34^{+}CD38^{+}$ cells enriched from cord blood $CD34^{+}$ cells.

Source data are provided in the Source Data file.

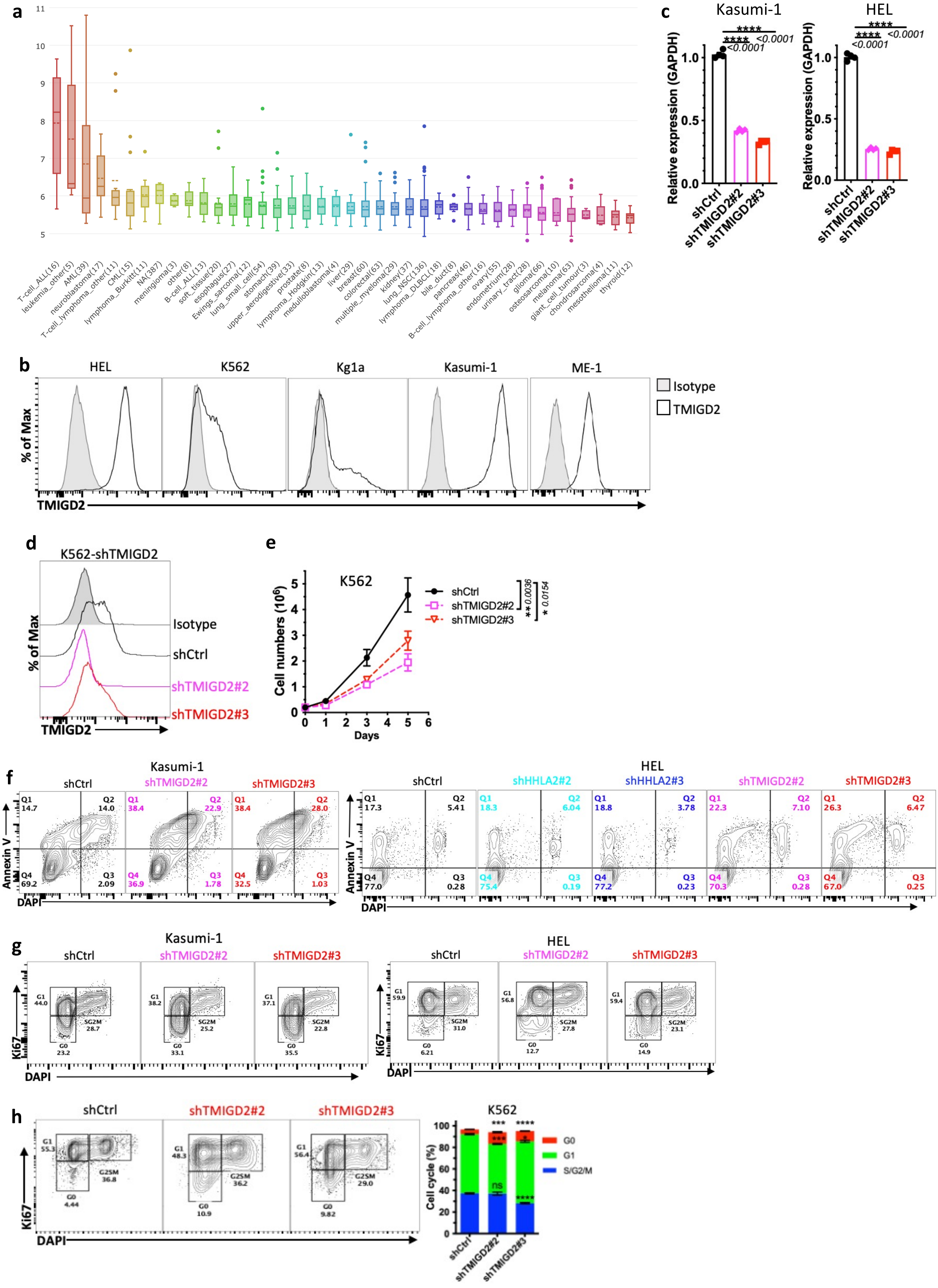

a
b
HEL
K562
Kg1a
Kasumi-1
ME-1
Isotype
TMIGD2
% of Max
c
Kasumi-1
HEL
Relative expression (GAPDH)
shCtrl
shTMIGD2#2
shTMIGD2#3
<0.0001
d
K562-shTMIGD2
e
K562
Cell numbers (10^6)
Days
f
Annexin V
DAPI
shHHLA2#2
shHHLA2#3
g
Ki67
h
Cell cycle (%)
G0
G1
S/G2/M

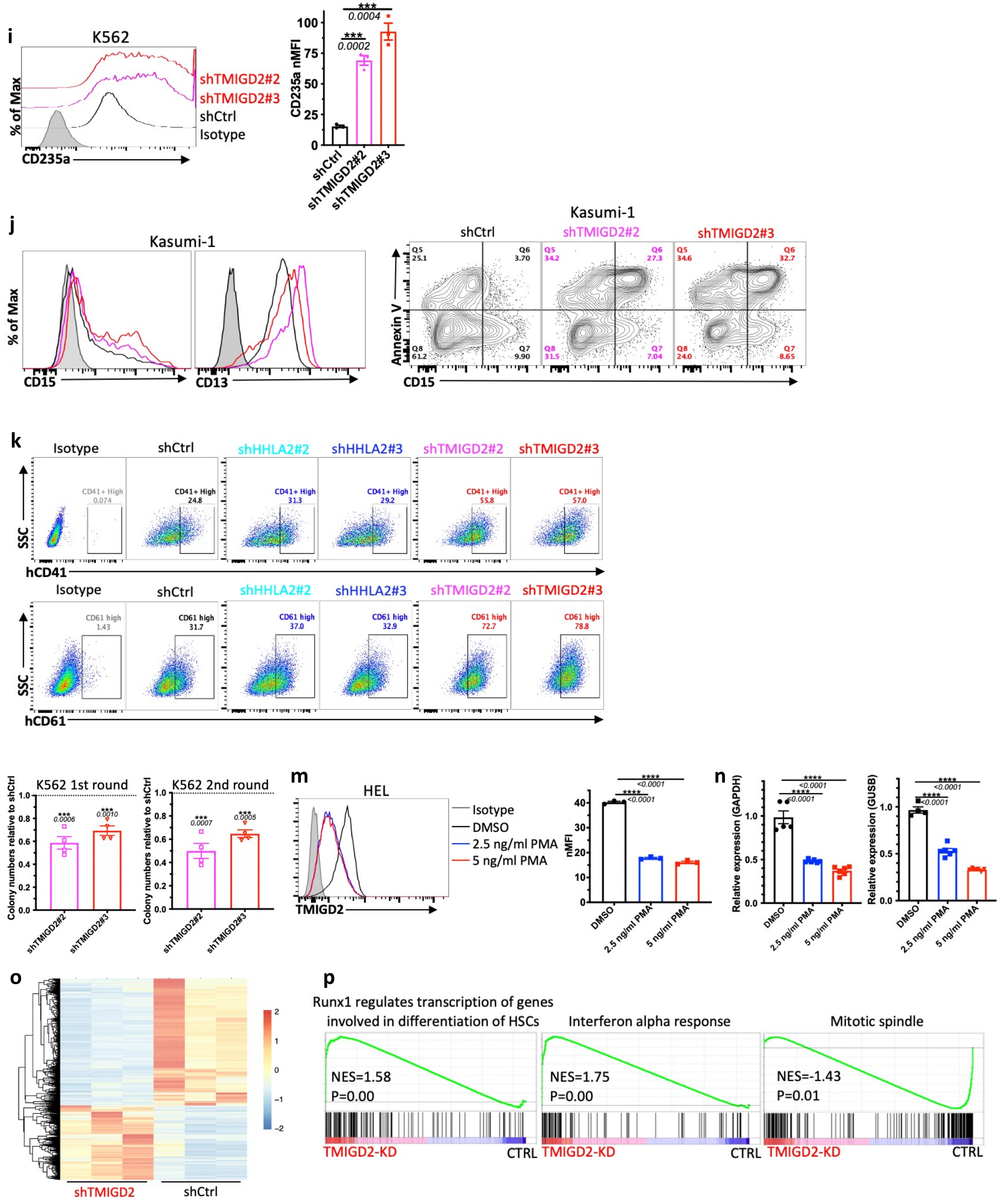
i
K562
% of Max
CD235a
shTMIGD2#2
shTMIGD2#3
shCtrl
Isotype
CD235a nMFI
***
0.0004
***
0.0002
shCtrl
shTMIGD2#2
shTMIGD2#3
j
Kasumi-1
% of Max
CD15
CD13
Kasumi-1
shCtrl
shTMIGD2#2
shTMIGD2#3
Annexin V
Q5 25.1
Q6 3.70
Q8 61.2
Q7 9.90
Q5 34.2
Q6 27.3
Q8 31.5
Q7 7.04
Q5 34.6
Q6 32.7
Q8 24.0
Q7 8.65
k
Isotype
shCtrl
shHHLA2#2
shHHLA2#3
shTMIGD2#2
shTMIGD2#3
SSC
hCD41
CD41+ High 0.074
CD41+ High 24.8
CD41+ High 31.3
CD41+ High 29.2
CD41+ High 55.8
CD41+ High 57.0
hCD61
CD61 high 1.43
CD61 high 31.7
CD61 high 37.0
CD61 high 32.9
CD61 high 72.7
CD61 high 78.8
l
K562 1st round
K562 2nd round
Colony numbers relative to shCtrl
*** 0.0006
*** 0.0010
*** 0.0007
*** 0.0005
shTMIGD2#2
shTMIGD2#3
m
HEL
Isotype
DMSO
2.5 ng/ml PMA
5 ng/ml PMA
TMIGD2
nMFI
**** <0.0001
**** <0.0001
DMSO
2.5 ng/ml PMA
5 ng/ml PMA
n
Relative expression (GAPDH)
Relative expression (GUSB)
**** <0.0001
**** <0.0001
o
shTMIGD2
shCtrl
p
Runx1 regulates transcription of genes involved in differentiation of HSCs
Interferon alpha response
Mitotic spindle
NES=1.58
P=0.00
NES=1.75
P=0.00
NES=-1.43
P=0.01
TMIGD2-KD
CTRL

Supplementary Fig. 2 | Roles of TMIGD2 in AML cells.

a, mRNA expression of TMIGD2 in malignant cell lines (n = 1036; CCLE, accessed in September 2013, Affymetrix).

b, Flow cytometry analysis of TMIGD2 expression on HEL, K562, Kg1a, Kasumi-1 and ME-1 human AML cell lines.

c, mRNA expression of TMIGD2 in control and TMIGD2 knockdown cells by qPCR.

d, K562 cells were transduced with shRNA targeting Ctrl and TMIGD2. K562 is endogenously HHLA2 negative.

e, Growth curves of K562 cells upon TMIGD2 knockdown.

f, Representative flow cytometry plots of apoptosis analysis in shCtrl, shHHLA2 and/or shTMIGD2 Kasumi-1 (left) and HEL (right) cells.

g, Representative flow cytometry plots of cell-cycle analysis in shCtrl and shTMIGD2 Kasumi-1 (left) and HEL (right) cells.

h, Cell-cycle analysis of K562 cells after TMIGD2 knockdown.

i, Representative flow cytometry plots (left) and statistics (right) showing the expression of differentiation marker CD235a on shCtrl and shTMIGD2 K562 cells.

j, Representative flow cytometry plots showing the expression of differentiation markers CD15 and CD13 (left), as well as co-expression of annexin V and CD15 (right) in shCtrl and shTMIGD2 Kasumi-1 cells.

k, Representative flow cytometry plots showing the expression of differentiation markers CD41 (up) and CD61 (down) on shCtrl, shHHLA2 and shTMIGD2 HEL cells.

l, CFC counts of K562 cells upon TMIGD2 knockdown.

m, Representative flow cytometry histogram (left) and statistics (right) of TMIGD2 expression on HEL cells after treatment with PMA for 48h. nMFI, mean fluorescence intensity relative to isotype control.

n, TMIGD2 mRNA level of HEL cells in **Extended Data Fig. 2m**. Housekeeping genes, GAPDH and GUSB.

o, Heatmap showing the overall differentially expressed genes in HEL cells with or without TMIGD2 knockdown.

p, GSEA of upregulated interferon alpha response and genes involved in differentiation of HSCs regulated by Runx1, as well as downregulated mitotic spindle pathway upon TMIGD2 knockdown. Mean ± SEM values are shown for **Extended Data Fig. 2**. *$p < 0.05$, **$p < 0.01$, ***$p < 0.001$, and ****$p < 0.0001$ by two-tailed Student's t test. Results are representative of three independent experiments. Color dots in **c**, **e**, **h**, **i**, and **l-n** represent technical replicates. Source data are provided in the Source Data file.

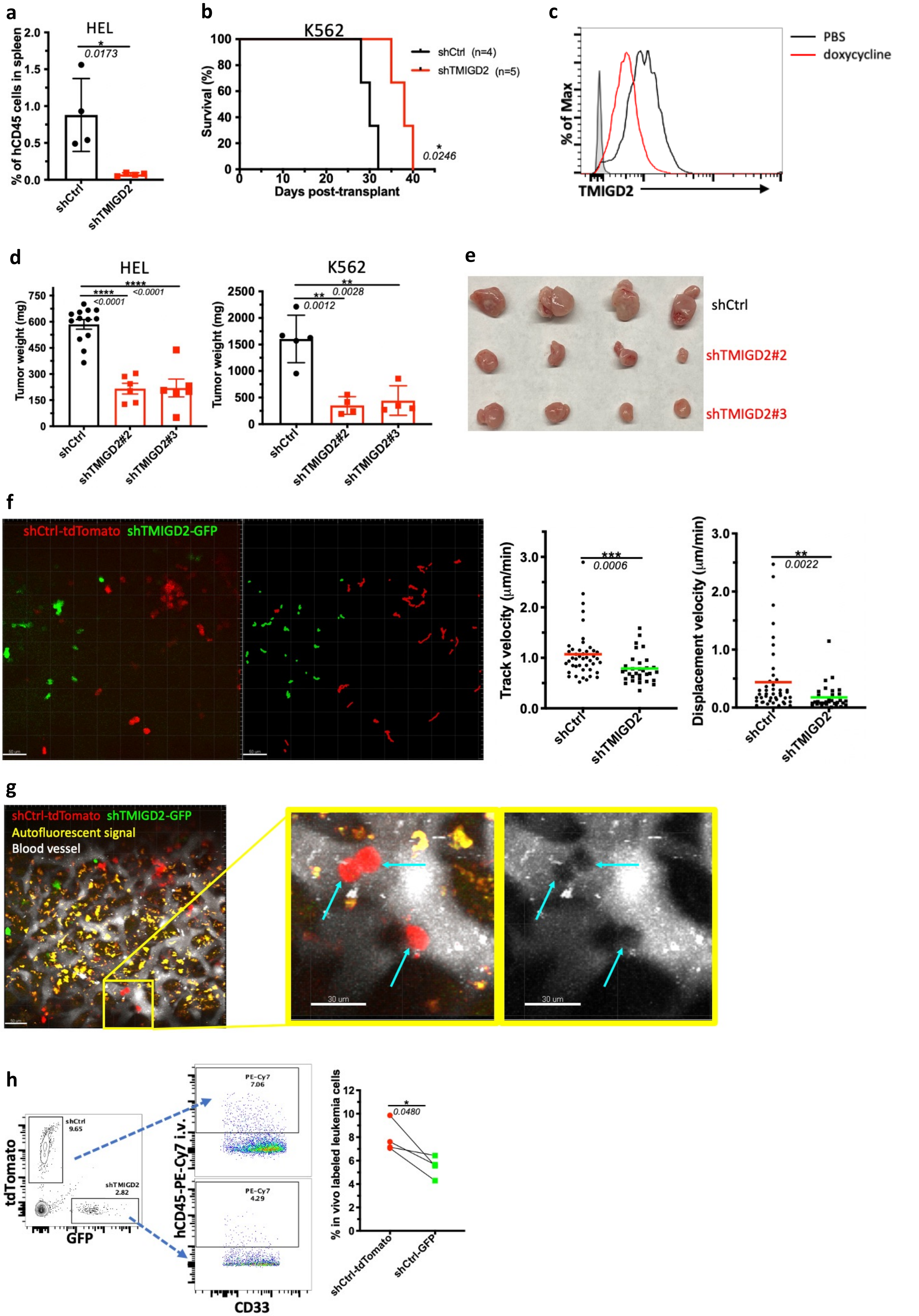
a
HEL
*
0.0173
% of hCD45 cells in spleen
2.0
1.5
1.0
0.5
0.0
shCtrl
shTMIGD2
b
K562
Survival (%)
100
80
60
40
20
0
0
10
20
30
40
Days post-transplant
shCtrl (n=4)
shTMIGD2 (n=5)
*
0.0246
c
% of Max
TMIGD2
PBS
doxycycline
d
HEL
Tumor weight (mg)
750
600
450
300
150
0
****
<0.0001
****
<0.0001
shCtrl
shTMIGD2#2
shTMIGD2#3
K562
Tumor weight (mg)
2500
2000
1500
1000
500
0
**
0.0012
**
0.0028
shCtrl
shTMIGD2#2
shTMIGD2#3
e
shCtrl
shTMIGD2#2
shTMIGD2#3
f
shCtrl-tdTomato shTMIGD2-GFP
Track velocity (μm/min)
3.0
2.0
1.0
0.0
***
0.0006
shCtrl
shTMIGD2
Displacement velocity (μm/min)
3.0
2.0
1.0
0.0
**
0.0022
shCtrl
shTMIGD2
g
shCtrl-tdTomato shTMIGD2-GFP
Autofluorescent signal
Blood vessel
30 um
30 um
h
tdTomato
GFP
shCtrl
9.65
shTMIGD2
2.82
hCD45-PE-Cy7 i.v.
CD33
PE-Cy7
7.06
PE-Cy7
4.29
% in vivo labeled leukemia cells
12
10
8
6
4
2
0
*
0.0480
shCtrl-tdTomato
shCtrl-GFP

Supplementary Fig. 3 | TMIGD2 depletion impairs AML development and dissemination.

a, Percentage of human leukemia cells in the spleen of NSG mice engrafted with shCtrl or shTMIGD2 HEL cells.

b, Kaplan-Meier survival curves of NSG mice receiving shCtrl (n=4) versus shTMIGD2 (n =5) K562 cells. The p value was calculated by the log-rank test.

c, Knockdown efficiency of TMIGD2 upon doxycycline treatment. The grey shaded histogram is isotype control for TMIGD2 staining.

d, Tumor weight of subcutaneous shCtrl and shTMIGD2 HEL (left)/ K562 (right) tumors in NSG mice.

e, Tumor volumes of subcutaneous shCtrl and shTMIGD2 HEL tumors in NSG mice.

f, Representative intravital image of tibial BM in XY showing shCtrl-tdTomato (red) and shTMIGD2-GFP (green) HEL cells engrafted with ratio of 1:7. Left, cell position and morphology at single time point; middle-left, cell tracks over time; middle-right, track velocity of both AML cells; right, displacement velocity of both AML cells. Scale bars, 50μm.

g, Representative intravital image of tibial BM in XY showing examples of shCtrl-tdTomato (red) HEL cells staying inside the vessels taking up vascular spaces that were not labeled by Qtracker 705 (indicated by arrow). Scale bars, 50μm (left) and 30μm (right, zoomed-in images).

h, Flow cytometry analysis of anti-hCD45-PE-Cy7 *in vivo* labeling of shCtrl-tdTomato and shTMIGD2-GFP HEL cells in the tibia BM.

Mean ± SD or SEM values are shown for **Extended Data Fig. 3**. *$p < 0.05$, **$p < 0.01$ and ***$p < 0.001$ by two-tailed Student's t test (**a**, **d** and **e**) or paired Student's t test (**g**). Results are representative of three independent experiments. Color dots in **f** represent different cells. Color dots in **a**, **d** and **h** represent individual mice. Source data are provided in the Source Data file.

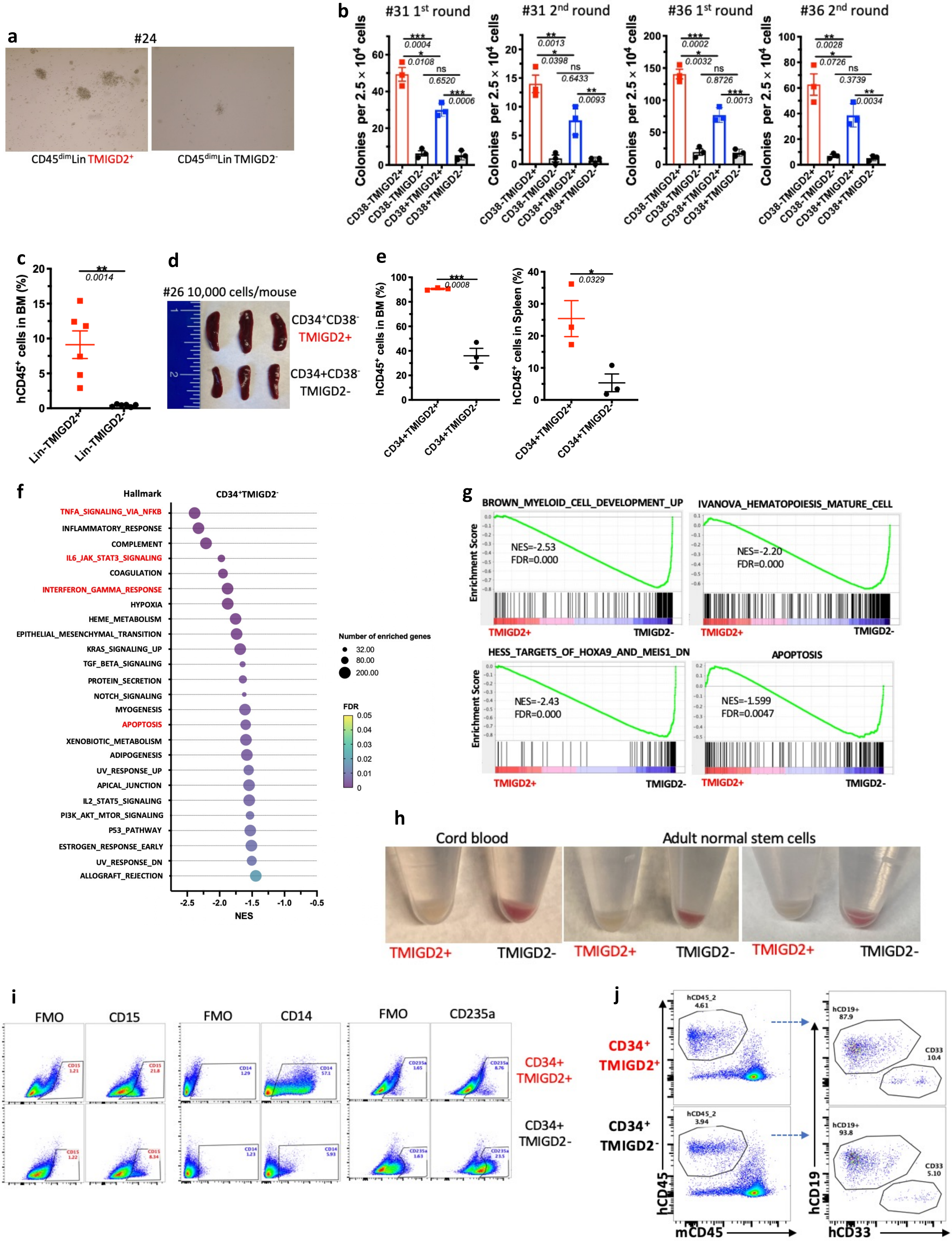

a
#24
CD45dimLin TMIGD2+
CD45dimLin TMIGD2-
b
#31 1st round
#31 2nd round
#36 1st round
#36 2nd round
Colonies per 2.5 × 10^4 cells
CD38-TMIGD2+
CD38-TMIGD2-
CD38+TMIGD2+
CD38+TMIGD2-
*** 0.0004
* 0.0108
ns 0.6520
*** 0.0006
** 0.0013
* 0.0398
ns 0.6433
** 0.0093
*** 0.0002
* 0.0032
ns 0.8726
*** 0.0013
** 0.0028
* 0.0726
ns 0.3739
** 0.0034
c
hCD45+ cells in BM (%)
** 0.0014
Lin-TMIGD2+
Lin-TMIGD2-
d
#26 10,000 cells/mouse
CD34+CD38- TMIGD2+
CD34+CD38- TMIGD2-
e
hCD45+ cells in BM (%)
*** 0.0008
hCD45+ cells in Spleen (%)
* 0.0329
CD34+TMIGD2+
CD34+TMIGD2-
f
Hallmark
CD34+TMIGD2-
TNFA_SIGNALING_VIA_NFKB
INFLAMMATORY_RESPONSE
COMPLEMENT
IL6_JAK_STAT3_SIGNALING
COAGULATION
INTERFERON_GAMMA_RESPONSE
HYPOXIA
HEME_METABOLISM
EPITHELIAL_MESENCHYMAL_TRANSITION
KRAS_SIGNALING_UP
TGF_BETA_SIGNALING
PROTEIN_SECRETION
NOTCH_SIGNALING
MYOGENESIS
APOPTOSIS
XENOBIOTIC_METABOLISM
ADIPOGENESIS
UV_RESPONSE_UP
APICAL_JUNCTION
IL2_STAT5_SIGNALING
PI3K_AKT_MTOR_SIGNALING
P53_PATHWAY
ESTROGEN_RESPONSE_EARLY
UV_RESPONSE_DN
ALLOGRAFT_REJECTION
NES
Number of enriched genes
32.00
80.00
200.00
FDR
g
BROWN_MYELOID_CELL_DEVELOPMENT_UP
NES=-2.53
FDR=0.000
IVANOVA_HEMATOPOIESIS_MATURE_CELL
NES=-2.20
FDR=0.000
HESS_TARGETS_OF_HOXA9_AND_MEIS1_DN
NES=-2.43
FDR=0.000
APOPTOSIS
NES=-1.599
FDR=0.0047
Enrichment Score
TMIGD2+
TMIGD2-
h
Cord blood
Adult normal stem cells
TMIGD2+
TMIGD2-
i
FMO
CD15
CD14
CD235a
CD34+ TMIGD2+
CD34+ TMIGD2-
j
hCD45_2 4.61
hCD45_2 3.94
hCD19+ 87.9
CD33 10.4
hCD19+ 93.8
CD33 5.10
CD34+ TMIGD2+
CD34+ TMIGD2-
hCD45
mCD45
hCD19
hCD33

Supplementary Fig. 4 | TMIGD2 is an enrichment surface marker for LSCs.

a, Representative colony images derived from $CD45^{dim}Lin^{-}TMIGD2^{+}$ and $CD45^{dim}Lin^{-}TMIGD2^{-}$ cells FACS-purified from Pt#24.

b, First round (left) and second round (right) CFC assays using FACS-purified $TMIGD2^{+}$ or $TMIGD2^{-}$ cells of $CD34^{+}CD38^{-}$ (CD38-) and $CD34^{+}CD38^{+}$ (CD38+) subpopulations from AML pt#31 and pt#36.

c, Representative engraftment potential of 3,000 FACS-purified $CD45^{dim}Lin^{-}TMIGD2^{+}$ or $CD45^{dim}Lin^{-}TMIGD2^{-}$ human leukemic cells in NSG-recipients three months post xenotransplantation in **Fig. 4d**.

d, Representative image of spleen from PDX mouse models engrafted with 10,000 FACS-purified $CD34^{+}CD38^{-}TMIGD2^{+}$ or $CD34^{+}CD38^{-}TMIGD2^{-}$ primary cells from AML pt#26.

e, Percentage of Leukemic cells in the BM (left) and spleen (right) from mice in **Extended Data Fig. 4d.**

f, Scattergrams of the top pathways that were significantly enriched in $CD34^{+}TMIGD2^{-}$ cells based on GSEA.

g, GSEA showed that the myeloid cell development pathway, hematopoiesis mature cell pathway, downregulated targets of *HOXA9* and *MEIS1*, and apoptosis pathway were significantly enriched in $CD34^{+}TMIGD2^{-}$ cells.

h, Representative photos of cell pellet from colonies derived from FACS-purified $TMIGD2^{+}$ or $TMIGD2^{-}$ subsets of $CD34^{+}$ HSPCs.

i, Representative flow cytometry plots showing the expression of CD14, CD15 and CD235a on cells from **Extended Data Fig. 4h**.

j, Representative Flow cytometry plots depicting the percentage of $hCD45^{+}$, $hCD33^{+}$ and $hCD19^{+}$ cells in the BM of NSG-recipients in **Fig. 4j**.

Mean ± SEM values are shown for **Extended Data Fig. 4**. *$p < 0.05$, **$p < 0.01$ and ***$p < 0.001$ by two-tailed Student's t test (**b, c** and **e**). Results are representative of three independent experiments. Color dots in **b** represent technical replicates. Color dots in **c** and **e** represent individual mice. Source data are provided in the Source Data file.

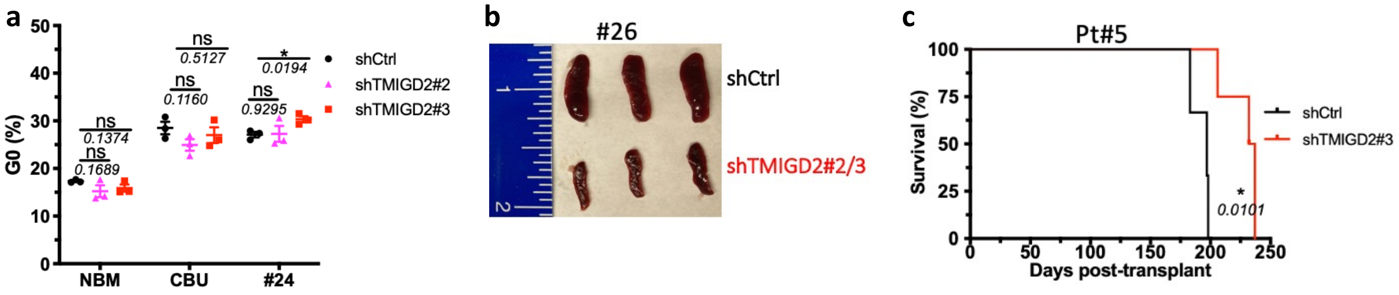

Supplementary Fig. 5 | TMIGD2 is required for leukemogenesis.

a, Percentage of G0 phase of cell cycle in shTMIGD2 compared with shCtrl primary $CD34^{+}$ cells as assessed by flow cytometry. Mean ± SEM values are shown. ns and *$p < 0.05$ by two-tailed Student's t test.

b, Representative image of spleen isolated from NSG-recipients in **Fig. 5f**.

c, Kaplan-Meier survival curves of AML PDX mouse models engrafted with primary shCtrl or shTMIGD2 AML cells (Pt#5). The p value was calculated by the log-rank test. Results are representative of three independent experiments. Color dots in **a** represent technical replicates. Source data are provided in the Source Data file.

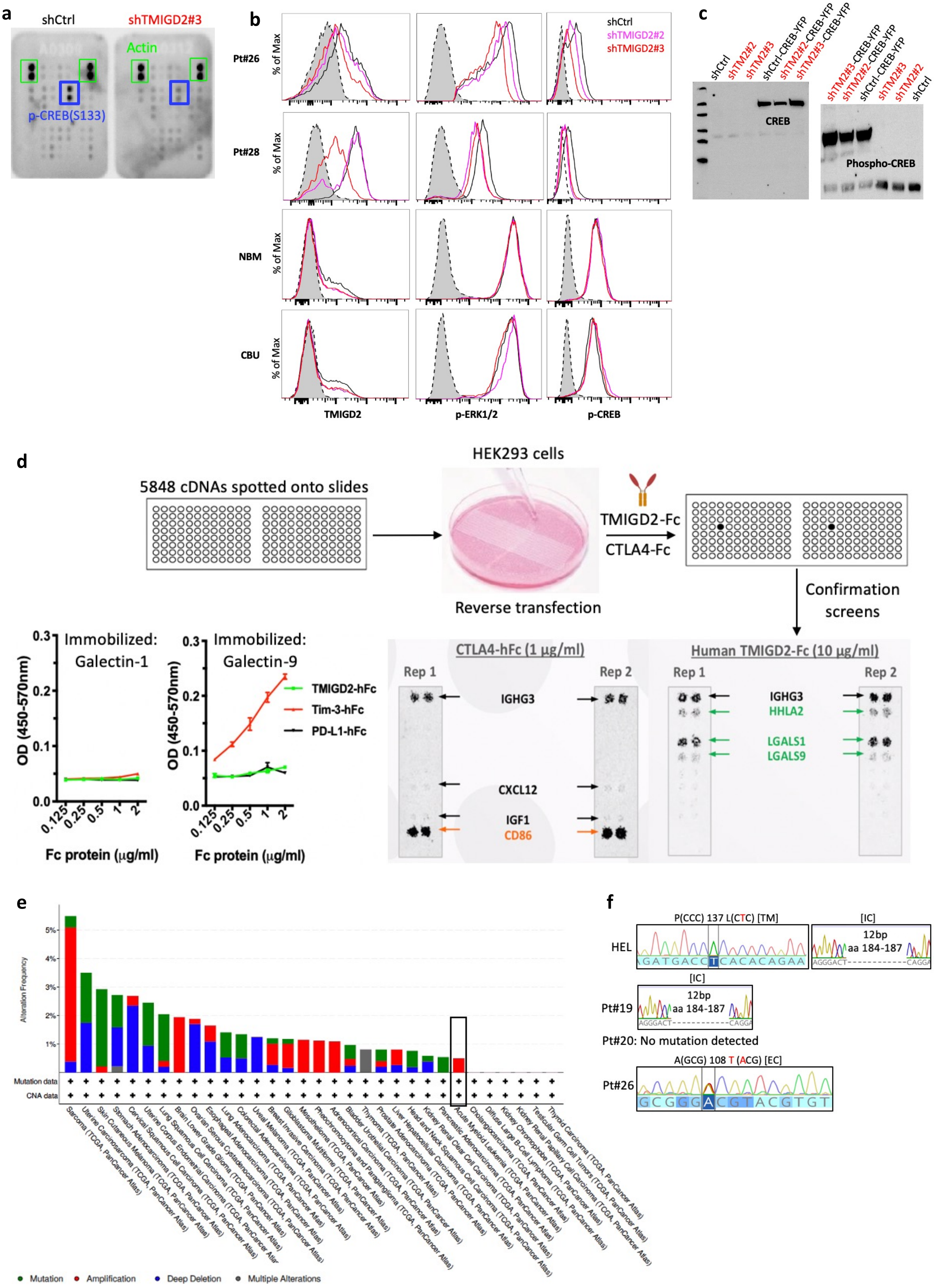

a
shCtrl
shTMIGD2#3
Actin
p-CREB(S133)
b
Pt#26
Pt#28
NBM
CBU
% of Max
TMIGD2
p-ERK1/2
p-CREB
shCtrl
shTMIGD2#2
shTMIGD2#3
c
shCtrl
shTM2#2
shTM2#3
shCtrl-CREB-YFP
shTM2#2-CREB-YFP
shTM2#3-CREB-YFP
CREB
Phospho-CREB
d
5848 cDNAs spotted onto slides
HEK293 cells
Reverse transfection
TMIGD2-Fc
CTLA4-Fc
Confirmation screens
Immobilized: Galectin-1
Immobilized: Galectin-9
OD (450-570nm)
Fc protein (µg/ml)
TMIGD2-hFc
Tim-3-hFc
PD-L1-hFc
CTLA4-hFc (1 µg/ml)
Human TMIGD2-Fc (10 µg/ml)
Rep 1
Rep 2
IGHG3
CXCL12
IGF1
CD86
HHLA2
LGALS1
LGALS9
e
Alteration Frequency
Mutation data
CNA data
Sarcoma (TCGA, PanCancer Atlas)
Uterine Carcinosarcoma (TCGA, PanCancer Atlas)
Skin Cutaneous Melanoma (TCGA, PanCancer Atlas)
Stomach Adenocarcinoma (TCGA, PanCancer Atlas)
Cervical Squamous Cell Carcinoma (TCGA, PanCancer Atlas)
Uterine Corpus Endometrial Carcinoma (TCGA, PanCancer Atlas)
Lung Squamous Cell Carcinoma (TCGA, PanCancer Atlas)
Brain Lower Grade Glioma (TCGA, PanCancer Atlas)
Ovarian Serous Cystadenocarcinoma (TCGA, PanCancer Atlas)
Esophageal Adenocarcinoma (TCGA, PanCancer Atlas)
Lung Adenocarcinoma (TCGA, PanCancer Atlas)
Colorectal Adenocarcinoma (TCGA, PanCancer Atlas)
Uveal Melanoma (TCGA, PanCancer Atlas)
Breast Invasive Carcinoma (TCGA, PanCancer Atlas)
Glioblastoma Multiforme (TCGA, PanCancer Atlas)
Mesothelioma (TCGA, PanCancer Atlas)
Pheochromocytoma and Paraganglioma (TCGA, PanCancer Atlas)
Adrenocortical Carcinoma (TCGA, PanCancer Atlas)
Bladder Urothelial Carcinoma (TCGA, PanCancer Atlas)
Thymoma (TCGA, PanCancer Atlas)
Prostate Adenocarcinoma (TCGA, PanCancer Atlas)
Liver Hepatocellular Carcinoma (TCGA, PanCancer Atlas)
Head and Neck Squamous Cell Carcinoma (TCGA, PanCancer Atlas)
Kidney Renal Clear Cell Carcinoma (TCGA, PanCancer Atlas)
Pancreatic Adenocarcinoma (TCGA, PanCancer Atlas)
Acute Myeloid Leukemia (TCGA, PanCancer Atlas)
Cholangiocarcinoma (TCGA, PanCancer Atlas)
Diffuse Large B-Cell Lymphoma (TCGA, PanCancer Atlas)
Kidney Chromophobe (TCGA, PanCancer Atlas)
Kidney Renal Papillary Cell Carcinoma (TCGA, PanCancer Atlas)
Testicular Germ Cell Tumors (TCGA, PanCancer Atlas)
Thyroid Carcinoma (TCGA, PanCancer Atlas)
Mutation
Amplification
Deep Deletion
Multiple Alterations
f
HEL
P(CCC) 137 L(CTC) [TM]
[IC]
12bp aa 184-187
Pt#19
Pt#20: No mutation detected
A(GCG) 108 T (ACG) [EC]
Pt#26

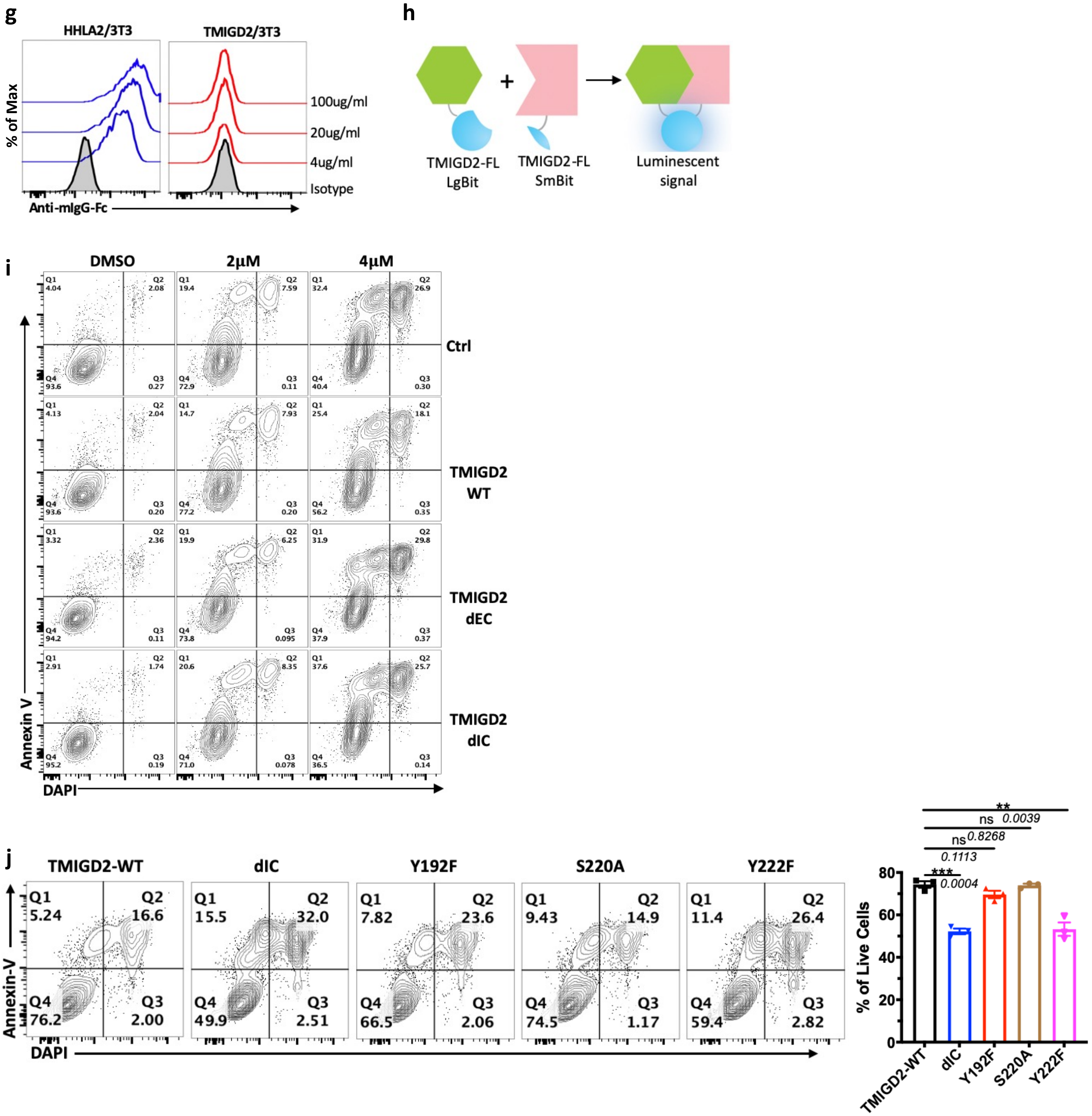

g
HHLA2/3T3
TMIGD2/3T3
% of Max
100ug/ml
20ug/ml
4ug/ml
Isotype
Anti-mIgG-Fc
h
+
TMIGD2-FL LgBit
TMIGD2-FL SmBit
Luminescent signal
i
DMSO
2μM
4μM
Ctrl
TMIGD2 WT
TMIGD2 dEC
TMIGD2 dIC
Annexin V
DAPI
Q1 4.04 Q2 2.08 Q4 93.6 Q3 0.27
Q1 19.4 Q2 7.59 Q4 72.9 Q3 0.11
Q1 32.4 Q2 26.9 Q4 40.4 Q3 0.30
Q1 4.13 Q2 2.04 Q4 93.6 Q3 0.20
Q1 14.7 Q2 7.93 Q4 77.2 Q3 0.20
Q1 25.4 Q2 18.1 Q4 56.2 Q3 0.35
Q1 3.32 Q2 2.36 Q4 94.2 Q3 0.11
Q1 19.9 Q2 6.25 Q4 73.8 Q3 0.095
Q1 31.9 Q2 29.8 Q4 37.9 Q3 0.37
Q1 2.91 Q2 1.74 Q4 95.2 Q3 0.19
Q1 20.6 Q2 8.35 Q4 71.0 Q3 0.078
Q1 37.6 Q2 25.7 Q4 36.5 Q3 0.14
j
TMIGD2-WT
dIC
Y192F
S220A
Y222F
Annexin-V
DAPI
Q1 5.24 Q2 16.6 Q4 76.2 Q3 2.00
Q1 15.5 Q2 32.0 Q4 49.9 Q3 2.51
Q1 7.82 Q2 23.6 Q4 66.5 Q3 2.06
Q1 9.43 Q2 14.9 Q4 74.5 Q3 1.17
Q1 11.4 Q2 26.4 Q4 59.4 Q3 2.82
% of Live Cells
0
20
40
60
80
TMIGD2-WT
dIC
Y192F
S220A
Y222F
***
0.0004
0.1113
ns
0.8268
ns
0.0039
**

Supplementary Fig. 6 | TMIGD2 supports AML via the ERK1/2-p90RSK-CREB signaling axis.

a, Human phospho-kinase array using shCtrl and shTMIGD2#3 HEL cell lysates.

b, Representative flow cytometry histogram showing the expression levels of TMIGD2, phospho-ERK1/2 (p-ERK1/2) and phospho-CREB (p-CREB) upon knockdown of TMIGD2 in primary $CD34^+$ cells isolated from NBM, CBU and AML patient samples (Pt#26 and Pt#28).

c, Western blot analysis of total CREB and phospho-CREB level in HEL cells engineered to express CREB with or without TMIGD2 knockdown.

d, Schematic for screening potential binding partners for TMIGD2 by high-throughput cell microarray. The binding of CTLA-4-hFc (left) and TMIGD2-hFc (right) to the target-expressing cells were shown. Plate-based binding assay with purified recombinant proteins showing no direct binding of TMIGD2 to Galectin-1 and Galectin-9. Tim-3-hFc protein was served as a positive control, which showed direct and specific binding with Galectin-9. PD-L1-hFc was used as a negative control.

e, TMIGD2 alteration frequency analysis by cBioPortal (http://www.cbioportal.org).

f, TMIGD2 mutation analysis by cDNA sequencing of three primary AML patient samples and HEL cell line.

g, Flow cytometry analysis showed that TMIGD2-mFc fusion protein bound with HHLA2/3T3 cells, but not TMIGD2/3T3 cells. 3T3 cells overexpressing HHLA2 or TMIGD2 were stained with TMIGD2-mFc fusion protein at different concentrations followed by incubation with anti-mIgGFc polyclonal antibody.

h, NanoBit proximity assay. TMIGD2-FL was fused to LgBit and SmBit and expressed in HEK 293 cells.

i, Representative flow cytometry plots of **Fig. 6h**.

j, Representative flow cytometry plots (left) and quantification (right) of live THP-1 cells upon 4µm 666-15 treatment. Tyrosine (Y) 192 and 222, as well as Serine (S) 220 of TMIGD2 were mutated to phenylalanine (F) and Alanine (A), respectively. Mean ± SEM values are shown. *$p < 0.05$, **$p < 0.01$ and ***$p < 0.001$ by two-tailed Student's t test. Results are representative of three independent experiments. Color dots in **j** represent technical replicates. Source data are provided in the Source Data file.

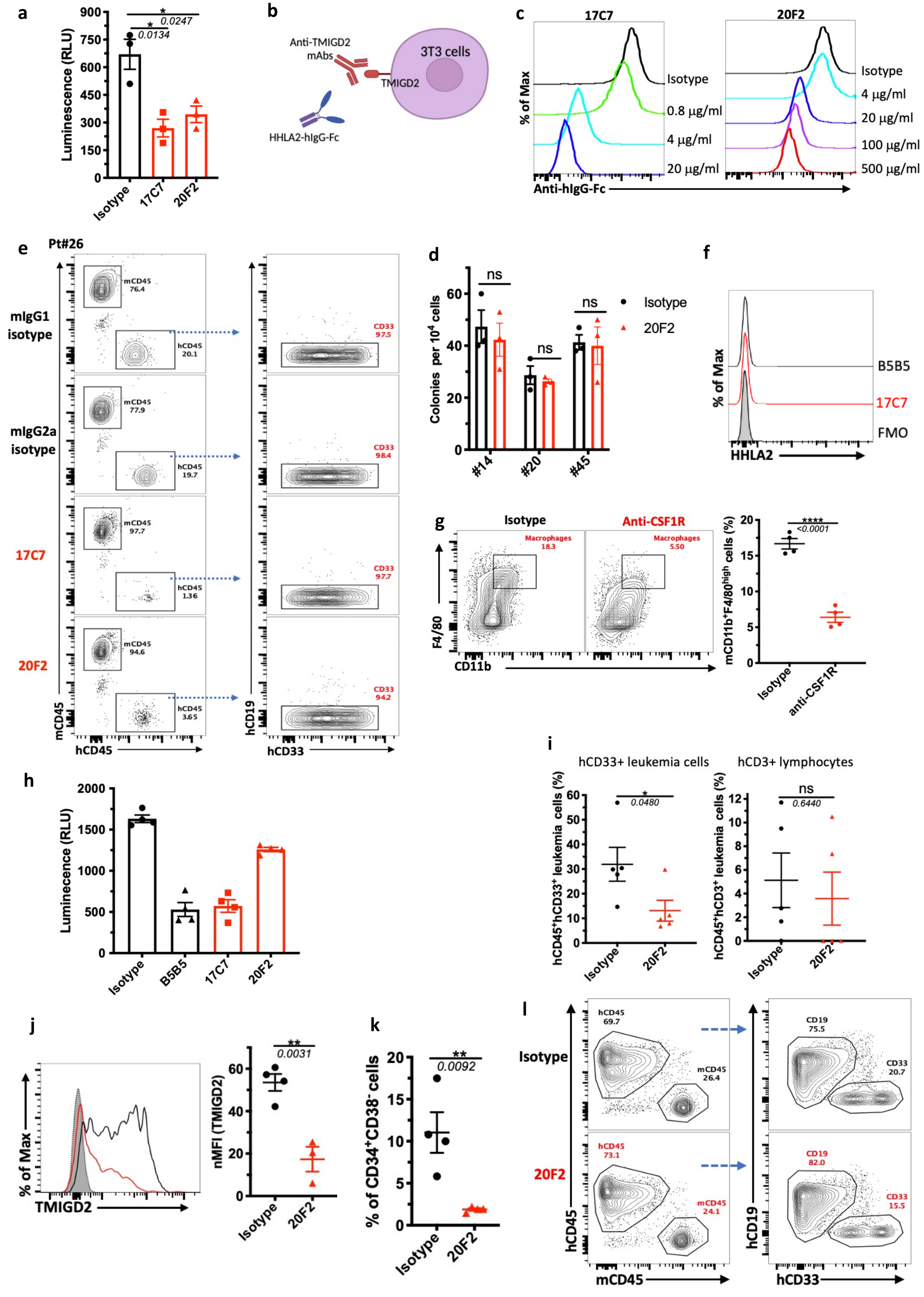
a
Luminescence (RLU)
900
750
600
450
300
150
0
*
0.0247
*
0.0134
Isotype
17C7
20F2
b
Anti-TMIGD2 mAbs
3T3 cells
TMIGD2
HHLA2-hIgG-Fc
c
17C7
20F2
% of Max
Isotype
0.8 μg/ml
4 μg/ml
20 μg/ml
Isotype
4 μg/ml
20 μg/ml
100 μg/ml
500 μg/ml
Anti-hIgG-Fc
e
Pt#26
mIgG1 isotype
mIgG2a isotype
17C7
20F2
mCD45 76.4
hCD45 20.1
CD33 97.5
mCD45 77.9
hCD45 19.7
CD33 98.4
mCD45 97.7
hCD45 1.36
CD33 97.7
mCD45 94.6
hCD45 3.65
CD33 94.2
mCD45
hCD45
hCD19
hCD33
d
Colonies per 10^4 cells
60
40
20
0
ns
ns
ns
Isotype
20F2
#14
#20
#45
f
% of Max
B5B5
17C7
FMO
HHLA2
g
Isotype
Anti-CSF1R
Macrophages 18.3
Macrophages 5.50
F4/80
CD11b
mCD11b+F4/80high cells (%)
20
15
10
5
0
****
<0.0001
Isotype
anti-CSF1R
h
Luminecence (RLU)
2000
1500
1000
500
0
Isotype
B5B5
17C7
20F2
i
hCD33+ leukemia cells
hCD3+ lymphocytes
hCD45+hCD33+ leukemia cells (%)
60
50
40
30
20
10
0
*
0.0480
Isotype
20F2
hCD45+hCD3+ leukemia cells (%)
12
10
8
6
4
2
0
ns
0.6440
Isotype
20F2
j
% of Max
TMIGD2
nMFI (TMIGD2)
60
40
20
0
**
0.0031
Isotype
20F2
k
% of CD34+CD38- cells
20
15
10
5
0
**
0.0092
Isotype
20F2
l
Isotype
20F2
hCD45 69.7
mCD45 26.4
CD19 75.5
CD33 20.7
hCD45 73.1
mCD45 24.1
CD19 82.0
CD33 15.5
hCD45
mCD45
hCD19
hCD33

Supplementary Fig. 7 | Anti-TMIGD2 mAbs inhibit AML *in vivo.*

a, NanoBit proximity assay showing anti-TMIGD2 mAbs (clones 17C7 and 20F2) disrupted the TMIGD2 *cis*-homodimerization.

b, **c** Experimental schematic (**b**) and flow cytometry plots (**c**) showing low concentration of 17C7 can completely block the interaction between HHLA2 and TMIGD2, while high concentration of 20F2 only partially block the HHLA2-TMIGD2 binding.

d, CFC counts of primary $TMIGD2^{-}CD34^{+}$ AML cells after treatment with isotype control (50μg/ml) or anti-TMIGD2 mAbs (20F2, 50μg/ml).

e, Representative flow cytometry plots showing the percentage of $hCD45^{+}CD33^{+}$ leukemia cells in the PB of AML PDX mouse model (Pt#26) after treatment with isotype controls or anti-TMIGD2 mAbs (17C7 and 20F2).

f, Representative flow cytometry plot showing the expression of HHLA2 on leukemia cells isolated from the BM of NSG-recipients in **Fig. 7e**.

g, Representative flow cytometry plots (left) and quantification (right) of macrophage percentages in the BM of NSG mice after four times of treatment with isotype control (rat IgG2a) or anti-CSF1R mAb.

h, Quantification of luminescence generated by adding Bio-Glo reagent into the coculture system. The TCR/CD3 effector (NFAT) cells were cocultured with HHLA2-expressing artificial APC cells in the presence of 10μg/ml B5B5, 17C7, 20F2 and isotype control.

i, Percentage of human $CD33^{+}$ and $CD3^{+}$ cells in the BM of NSG mice engrafted with mononuclear cells, including immune cells and leukemia cells, from AML patient.

j, Representative flow cytometry histogram (left) and quantification (right) of TMIGD2 expression on BM $hCD33^{+}$ cells isolated from PDX mice which were treated with isotype control or 20F2 mAb.

k, Percentage of $CD34^{+}CD38^{-}$ cells isolated from PDX mice which were treated with isotype control or 20F2 mAb.

l, Representative flow cytometry plots showing the percentage of human $CD45^{+}$, $CD33^{+}$ and $CD19^{+}$ cells in the BM of NSG mice from **Fig. 7k**.

Mean ± SEM values are shown for **Extended Data Fig. 7**. $^{**}p < 0.01$ and $^{****}p < 0.0001$ by two-tailed Student's t test. Results are representative of three independent experiments. Color dots in **a**, **d** and **h** represent technical replicates. Color dots in **g**, **i**, **j** and **k** represent individual mice. Source data are provided in the Source Data file.

Supplementary Table 1. Patient characteristics (n=41) of AML samples used for flow cytometry analysis, Related to Fig. 1.

Age at diagnosis	
Median	61
Range	28-94
White blood cell count ($\times 10^9$/L)	
Median	62.4
Range	1.6-197
CD34 count (%)	
Median	36.2
Range	0.11-99.1
Risk group according to ELN (no. (%))	
Intermediate	10 (23%)
Unfavorable	10 (23%)
U.D.	24 (54%)
FLT3 (no. (%))	
WT	11 (25%)
ITD	8 (18%)
U.D.	25 (57%)
NPM1 (no. (%))	
WT	17 (39%)
Mutant	6 (14%)
U.D.	21 (47%)
Cytogenetic characteristics (no. (%))	
Normal	19 (43%)
t(8;21)	1 (2%)
Inv(16)	2 (5%)
del(5q)	1 (2%)
Other	2 (5%)
U.D.	19 (43%)

ITD, internal tandem duplication
WT, wild-type
U.D., Undetermined

Supplementary Table 2. Clinical characteristics of primary specimens between TMIGD2 high and TMIGD2 low cohorts, Related to Fig. 1.

TMIGD2 Level	Patient numbers	<5% of $CD34^+$ cells	<15% of $CD34^+$ cells	NPM1 mutation	Mutated NPM1 with FLT3-ITD$^{high^}$	ELN risk		
						Favorable	Intermediate	Adverse
High (>50%)	26	(11.5%) 3/26	(26.9%) 7/26	(8.3%) 1/12*	(0%) 0/1	N/A	4	6
Low (<50%)	17	(52.9%) 9/17	(58.8%) 10/17	(77.8%) 7/9*	(57.1%) 4/7	N/A	6	4

^ High allelic ratio (>0.5)
*Clinical characteristics available

Supplementary Table 3. Clinical characteristics of primary AML specimens used for CFC assays and engraftment experiments in this study.

Sample ID	Sex	De Novo/ Relapsed	Risk group according to ELN	FLT3	Cytogenetic /Mutations	WBC count
**#21**	F	New dx	Intermediate. Risk score: 1.22	N/A	Normal cytogenetics. Mutations N/A	87,000
**#24**	F	New dx	Intermediate. Risk score: 0.634	Negative	Favorable. CBF-AML, RUNX1T1/RUNX1 fusion with the t(8;21)(q22;q22.3) translocation	29,000
**#27**	N/A	N/A	N/A	N/A	N/A	N/A
**#28**	M	R/R AML	N/A	FLT-3 positive (acquired after relapse)	FLT3.IDH2.RUNX1.TET2	152,700
**#31**	F	Relapsed AML/ after AlloSCT	Unfavorable. Risk score: 1.327	FLT-3 ITD ratio > 1	NPM1, DNMT3A and WT1 mutations, 47, XX, add (18)(p11.2), +22[20]	168,000
**#36**	M	New dx	Intermediate. Risk score: 1.186	Negative	Favorable. INV 16. CBFB	89,000
**#40**	M	R/R AML	Intermediate. Risk score: 0.925	FLT3-ITD ratio 0.78	NPM1. WT1	31,200
**#14**	F	New dx	Intermediate. Risk score: 1.22	N/A	Normal cytogenetics. Mutations N/A	87,000
**#20**	M	New dx	Unfavorable. Risk score: 2.154	FLT3-ITD	CEBPA. DNMT3A. NPM1	94,400
**#45**	F	Relapsed	N/A	N/A	RUNX1, KIT, SF3B1, EZH2, GNAS	72,400
**#8**	M	New dx	Unfavorable. Risk score: 1.578	negative	CEBPA(2). EZH2. NF1(2). RUNX1, SMC1A. TET2(2)	75,100
**#26**	M	Secondary (had Myeloma)	N/A	N/A	DNMT3A.GNAS.KIT. NRAS. SETBP1	1,600
**#5**	M	New dx	Unfavorable. Risk score: 1.489	Negative	KIT, WT1, PTPN11. Abnormal. High Risk.49, XY, +8, inv(16)(p13.1q22), +21.+22[14]/46,XY[5]	134,000
**#19**	M	New dx	N/A	negative	PHF6.RUNX1	12,900

Dx, diagnosis. WBC, white blood cell count. N/A, not available. R/R, relapsed/refractory. AlloSCT, allogeneic stem cell transplantation. Age at diagnosis: median, 61; age range, 28-94.

Supplementary Table 4. Key resources

REAGENT or RESOURCE	SOURCE	IDENTIFIER
**Antibodies**		
Anti-human CD3-BV421 (Clone UCHT1) (1: 250)	BD	Cat#562427; RRID: AB_11152082
Anti-human CD11b-APC (Clone ICRF44) (1: 250)	Biolgend	Cat#301309; RRID: AB_314161
Anti-human CD14-Alexa 700 (Clone HCD14) (1: 200)	Biolgend	Cat#356608; RRID: AB_2561904
Anti-human CD15-FITC (HI98) (1: 250)	Biolgend	Cat#301903; RRID: AB_314195
Anti-human CD19-Alexa 700 (clone HIB19) (1: 250)	Biolgend	Cat#325614; RRID: AB_830687
Anti-human CD19-PE/Cy7 (Clone HIB19) (1: 250)	Biolgend	Cat#982410; RRID: AB_2715770
Anti-human CD33-PE (Clone WM53) (1: 250)	Biolgend	Cat#303404; RRID: AB_314348
Anti-human CD34-APC (Clone 581) (1: 200)	Biolgend	Cat#343510; RRID: AB_1877153
Anti-human CD38-PE/Cy7 (Clone HB-7) (1: 250)	Biolgend	Cat#302226; RRID: AB_493751
Anti-human CD41-PE (Clone HIP8) (1: 250)	Biolgend	Cat#303705; RRID: AB_314375
Anti-human CD45-BUV395 (Clone HI30) (1: 250)	BD	Cat#563792; RRID: AB_2869519
Anti-human CD45-FITC (Clone HI30) (1: 250)	Biolgend	Cat#982316; RRID: AB_2876779
Anti-human CD45-PE/Cy7 (Clone HI30) (1: 250)	Biolgend	Cat#304015; RRID: AB_314403
Anti-human CD45RA-FITC (Clone HI100) (1: 250)	Biolgend	Cat#304106; RRID: AB_314410
Anti-human CD61-APC (Clone VI-PL2) (1: 250)	Biolgend	Cat#336411; RRID: AB_10707694
Anti-human CD123-PE/Dazzle (Clone 6H6) (1: 250)	Biolgend	Cat#306034; RRID: AB_2566450
Anti-human CD235a-PE (Clone HI264) (1: 250)	Biolgend	Cat#349105; RRID: AB_10641707
Anti-human IL1RAP-PE (Clone 89412) (1: 250)	R&D	Cat#FAB676P; RRID: AB_10717521
Anti-human IgG Fc-PE (Clone HP6017) (1: 250)	Biolgend	Cat#409303; RRID: AB_10900424
Anti-human IgG Fc-APC (Clone HP6017) (1: 250)	Biolgend	Cat#409306; RRID: AB_11150591
Anti-mouse CD11b-APC (Clone M1/70) (1: 250)	Biolgend	Cat#101208; RRID: AB_312791
Anti-mouse F4/80-APC (Clone BM8) (1: 250)	Biolgend	Cat#123115; RRID: AB_893493
Anti-mouse CD45-APC (Clone 30-F11) (1: 250)	Biolgend	Cat#103112; RRID: AB_312977
Anti-mouse CD45-Alexa 700 (Clone 30-F11) (1: 250)	Biolgend	Cat#103128; RRID: AB_493715
Goat anti-mouse IgG APC (polyclonal antibody) (1: 250)	eBioscience	Cat#17401082; RRID: AB_2573203
Goat anti-mouse IgG PE (polyclonal antibody) (1: 250)	Biolgend	Cat#405307; RRID: AB_315010
Anti-human TMIGD2 (Clone 953743) (2 μg/ml)	R&D	Cat#MAB83162100
Anti-human Ki-67-APC (Clone Ki-67) (1: 250)	Biolgend	Cat#350513; RRID: AB_10959326
Anti-ERK1/2 Phospho-PE/Cy7 (Clone 6B8B69) (1: 250)	Biolgend	Cat#369515; RRID: AB_2721653
Phospho-CREB (Ser133)-AF647 (Clone 87G3) (1: 250)	Cell Signaling Technology	Cat#14001; RRID: AB_2798359
Anti-human TMIGD2-PE (Clone 17C7) (5 μg/ml)	This paper	N/A
Anti-human TMIGD2 (Clone 17C7) (2 μg/ml)	This paper	N/A
Anti-human TMIGD2 (Clone 20F2) (2 μg/ml)	This paper	N/A
Anti-human HHLA2 (Clones B5B5, A3H11) (2 μg/ml)	This paper	N/A
Anti-β-actin (Clone C11) (1:5 000)	Santa Cruz	Cat#sc-1615; RRID: AB_630835
Anti-phospho ERK1/2 (Thr202/Tyr204) (Clone 6B8B69) (1: 2000)	Biolgend	Cat#369502; RRID: AB_2721735
Anti-total ERK (Clone 137F5) (1: 2000)	Cell Signaling Technology	Cat#4695T; RRID: AB_2339400

Anti-phospho Akt (Ser473) (Clone D9E) (1: 2000)	Cell Signaling Technology	Cat#4060T; RRID: AB_2315049
Anti-total Akt (clone C67E7) (1: 2000)	Cell Signaling Technology	Cat#4691T; RRID: AB_915783
Anti-total CREB (A18233D) (1: 2000)	Biolgend	Cat#948301; RRID: AB_ 2894537
Anti-phospho CREB (Ser133) (Clone 87G3) (1: 2000)	Cell Signaling Technology	Cat#9198S; RRID: AB_2561044
Goat anti-rabbit IgG-HRP (1: 5000)	Cell Signaling Technology	Cat#7074S; RRID: AB_2099233
Goat anti-mouse IgG-HRP (1: 5000)	Jackson Immuno-Research	Cat#115035003; RRID: AB_10015289
Rabbit anti-goat IgG-HRP (1: 5000)	Jackson Immuno-Research	Cat#305035003; RRID: AB_2339400
Goat anti-human IgG-HRP (1: 5000)	Southern Biotech	Cat#2040-05; RRID: AB_2795644
Anti-phospho p90RSK (Thr359/Ser363) (1: 2000)	Cell Signaling Technology	Cat#9344; RRID: AB_331650
Bcl-2 (Clone 124) (1: 2000)	Cell Signaling Technology	Cat#15071; RRID: AB_2744528
InVivoMab anti-mouse CSF1R (Clone AFS98)	Bio X Cell	Cat#BE0213; RRID: AB_2687699
InVivoMab rat IgG2a isotype control (Clone 2A3)	Bio X Cell	Cat#BE0089; RRID: AB_1107769
Mouse IgG1 isotype control (Clone HKSP)	Leinco Technologies	Cat#I-536; RRID: AB_2737545
Mouse IgG2a isotype control (Clone C1.18.4)	Leinco Technologies	Cat#I-118; RRID: AB_2737531
**Bacterial and Virus Strains**		
NEB 5-alpha Competent *E. coli* (high efficiency)	NEB	Cat#C2987H
**Biological Samples**		
Cord blood units	New York Blood Center, USA	N/A
Peripheral blood/bone marrow samples from AML patients; Adult normal bone marrow samples	Montefiore Medical Center, Bronx, USA.	Approved by the Einstein IRB to Dr. Sica: 11-02-060E
**Chemicals, Peptides, and Recombinant Proteins**		
CFSE	Invitrogen	Cat#C34554
Qtracker 705	Invitrogen	Cat#Q21061MP
DAPI	Biolgend	Cat#422801
Annexin V-APC	Biolgend	Cat#640920
Protease and Phosphatase Inhibitor Cocktail (100X)	Thermo Scientific	Cat#78440
Human HHLA2-hFc	R&D	Cat#8084-B7-050
Human TMIGD2 EC-mIgG-Fc	This paper	N/A
Human TMIGD2 EC-hIgG-Fc	This paper	N/A

Human TMIGD2-hFc	R&D	Cat#8316-TR-500
Human Tim-3-hFc	R&D	Cat#2365-TM-050
Human PD-L1-hFc	R&D	Cat#156-B7-100
Human Galectin-1	R&D	Cat#1152-GA-050/CF
Human Galectin-9	R&D	Cat#2045-GA-050
Protein G Resin	GenScript	Cat#L00209
Polybrene	Merck Millipore	Cat#TR-1003-G
Doxycycline Hyclate<br><br>Promega Bio-Glo™ Luciferase Assay System	Santa Cruz Biotechnology<br>Promega	Cat#sc-204734<br><br>Cat#PRG7940
TMB substrate solution	Thermo Scientific	Cat#N301
**Critical Commercial Assays**		
SiteClickTM R-PE Antibody Labeling Kit	Invitrogen	Cat#S10467
jetPRIME®, DNA and siRNA Transfection Reagent	Polyplus Transfection	Cat#114-07
ZymoPURE II Plasmid Midiprep Kit	Zymo Research	Cat#D4200
Gibson Assembly Master Mix	NEB	Cat#E2611S
Q5® Site-Directed Mutagenesis Kit	NEB	Cat#E0554S
Human CD34 MicroBead Kit	Miltenyi Biotec	Cat#130-046-702
RNeasy Plus Micro Kit	QIAGEN	Cat#74034
RNeasy Mini Kit	QIAGEN	Cat#74104
Human Phospho-Kinase Array Kit	R&D	Cat#ARY003B
Clarity™ Western ECL Substrate	Bio-Rad	Cat#1705060
T Cell Activation Bioassay	Promega	Cat#J1601
**Experimental Models: Cell Lines**		
TMIGD2 EC-hIgG/S2 cell	This paper	N/A
TMIGD2 EC-mIgG/S2 cell	This paper	N/A
Mouse: NIH-3T3 cell	ATCC	Cat#CRL-1658; RRID: CVCL_0594
TMIGD2/3T3 cell	This paper	
HHLA2/3T3 cell	Zang lab	Zhao et al., 2013
Human: Phoenix-AMPHO cell	ATCC	Cat#CRL-3213; RRID: CVCL_H716
HEK 293T cell	Gift from Dr. Wenjun Guo	N/A
Human: K562 cell	ATCC	Cat#CCL-243; RRID: CVCL0004
Human: HEL cell	Gift from Dr. Ulrich Steidl	N/A
shHHLA2 HEL cell	This paper	N/A
shTMIGD2 HEL/Kasumi-1 cell	This paper	N/A
shTMIGD2 K562 cell	This paper	N/A
THP-1 cell	Gift from Dr. Ulrich Steidl	N/A
TMIGD2-FL/THP-1 cell	This paper	N/A
TMIGD2-dEC/THP-1 cell	This paper	N/A
TMIGD2-dIC/THP-1 cell	This paper	N/A

Kg1a, ME-1, Kasumi-1 cells	Gift from Dr. Ulrich Steidl	N/A
**Experimental Models: Organisms/Strains**		
NSG mice	The Jackson Laboratory	Cat#005557; RRID: IMSR_JAX:005557
BALB/c mice	Charles River Laboratory	Cat#CRL:028; RRID: IMSR_CRL:028
**Recombinant DNA**		
pMT/Bip-TMIGD2 EC-mIgGFc	This paper	N/A
pMT/Bip-TMIGD2 EC-hIgGFc	This paper	N/A
MSCV-TMIGD2-FL-IRES-YFP	This paper	N/A
MSCV-TMIGD2-dEC-IRES-YFP	This paper	N/A
MSCV-TMIGD2-dIC-IRES-YFP	This paper	N/A
pCDH-EF1-Luc2-P2A-tdTomato	Gift from Dr. Wenjun Guo	N/A
pSIH1-H1-copGFP shRNA	Gift from Dr. Ulrich Steidl	N/A
pLKO.1 GFP shRNA	Gift from David Sabatini	Addgene plasmid #30323; RRID: Addgene_30323
pLKO-Tet-On	Gift from Dmitri Wiederschain	Addgene plasmid #21915. RRID: Addgene_21915
shHHLA2	This paper	N/A
shTMIGD2	This paper	N/A
pCMV-VSV-G	Gift from Bob Weinberg	Addgene Cat#8454; RRID: Addgene_8454
psPAX2	Gift from Didier Trono	Addgene plasmid #12260; RRID: Addgene_12260
**Software and Algorithms**		
GraphPad Prism version 9	GraphPad Software	https://www.graphpad.com
FlowJo version 10.8	BD	https://www.flowjo.com
Image Lab 6.0.0	Bio-Rad	https://www.bio-rad.com
L-Calc software	Stemcell technology	https://www.stemcell.com/l-calc-software.html
Imaris 9.3	Bitplane	N/A
R version 3.6.2	R Core	https://cran.r-project.org
DESeq2 (Version 3.11)	Love et al., 2014	https://bioconductor.org/packages/release/bioc/html/DESeq2.ht ml
GSEA	Subramanian et al., 2005	https://www.gsea-msigdb.org/gsea/index.jsp
**Other**		
Wallac 1420 Victor2 Microplate Reader	Perkin Elmer	N/A
ChemiDocTM Touch Imaging System	Bio-Rad	N/A
Olympus FVE-1200 upright microscope	Olympus	N/A
Deepsee MaiTai Ti-Sapphire pulsed laser	Spectra-Physics	N/A
