# Supplementary material for: TMIGD2 is an orchestrator and therapeutic target on human acute myeloid leukemia stem cells
Source: Nat Commun. 2024 Jan 2;15:11. doi: 10.1038/s41467-023-43843-6 (PMC10761673; doi:10.1038/s41467-023-43843-6)
Supplement: Supplementary file 3 — Reporting Summary [file 41467_2023_43843_MOESM3_ESM.pdf]

nature portfolio

Corresponding author(s): Xingxing Zang

Last updated by author(s): Oct 23, 2023

Reporting Summary

Nature Portfolio wishes to improve the reproducibility of the work that we publish. This form provides structure for consistency and transparency in reporting. For further information on Nature Portfolio policies, see our Editorial Policies and the Editorial Policy Checklist.

Statistics

For all statistical analyses, confirm that the following items are present in the figure legend, table legend, main text, or Methods section.

n/a	Confirmed	
☐	☒	The exact sample size (*n*) for each experimental group/condition, given as a discrete number and unit of measurement
☐	☒	A statement on whether measurements were taken from distinct samples or whether the same sample was measured repeatedly
☐	☒	The statistical test(s) used AND whether they are one- or two-sided<br>*Only common tests should be described solely by name; describe more complex techniques in the Methods section.*
☐	☒	A description of all covariates tested
☐	☒	A description of any assumptions or corrections, such as tests of normality and adjustment for multiple comparisons
☐	☒	A full description of the statistical parameters including central tendency (e.g. means) or other basic estimates (e.g. regression coefficient) AND variation (e.g. standard deviation) or associated estimates of uncertainty (e.g. confidence intervals)
☐	☒	For null hypothesis testing, the test statistic (e.g. *F*, *t*, *r*) with confidence intervals, effect sizes, degrees of freedom and *P* value noted<br>*Give P values as exact values whenever suitable.*
☒	☐	For Bayesian analysis, information on the choice of priors and Markov chain Monte Carlo settings
☒	☐	For hierarchical and complex designs, identification of the appropriate level for tests and full reporting of outcomes
☒	☐	Estimates of effect sizes (e.g. Cohen's *d*, Pearson's *r*), indicating how they were calculated

Our web collection on statistics for biologists contains articles on many of the points above.

Software and code

Policy information about availability of computer code

Data collection	BD FACS Diva 8.0 for flow cytometry acquisition, Olympus FVE-1200 upright microscope for intravital imaging, Wallac 1420 Victor2 Microplate Reader for Elisa and Nanobit assay, ChemiDoc Imaging System (Bio-Rad) for western blot, Caliper Life Sciences IVIS Spectrum intravital imaging system, ABI QuantStudio 6 Pro Real -Time PCR System for qPCR.
Data analysis	Statistical analyses were performed using GrapPad Prism 9 (GraphPad Software, Inc.). Two-tailed Student's t test or paired Student's t test was used to compare means between groups as indicated; p < 0.05 was considered statistically significant. Kaplan-Meier survival curves were plotted with GraphPad Prism 9 and the p values were calculated using the log-rank (Mantel Cox) test. GSEA 4.1.0 for GSEA analysis. Flowjo 10.8.1 for flow cytometry analysis.

For manuscripts utilizing custom algorithms or software that are central to the research but not yet described in published literature, software must be made available to editors and reviewers. We strongly encourage code deposition in a community repository (e.g. GitHub). See the Nature Portfolio guidelines for submitting code & software for further information.

Data

Policy information about availability of data

All manuscripts must include a data availability statement. This statement should provide the following information, where applicable:

- Accession codes, unique identifiers, or web links for publicly available datasets
- A description of any restrictions on data availability
- For clinical datasets or third party data, please ensure that the statement adheres to our policy

RNA-seq data that support Fig. 2 and Fig. 4 has been deposited in the Gene Expression Omnibus (GEO), with accession numbers GSE214480 and GSE214487, respectively. Supplementary Fig. 1a was generated using BloodSpot database (www.bloodspot.eu). Supplementary Fig. 1d, 2a, and 6e were generated using data from cBioPortal (https://www.cbioportal.org/).

Research involving human participants, their data, or biological material

Policy information about studies with human participants or human data. See also policy information about sex, gender (identity/presentation), and sexual orientation and race, ethnicity and racism.

Reporting on sex and gender	Sex and gender were not considered when collecting patient samples.
Reporting on race, ethnicity, or other socially relevant groupings	Race, ethnicity, or other socially relevant groupings were not considered.
Population characteristics	Age median 61. Range 28-94.
Recruitment	Patient samples were randomly collected.
Ethics oversight	Human AML samples and healthy donor samples were obtained with informed consent at Montefiore Medical Center/Albert Einstein Cancer Center in congruence with the protocol approved by the institutional review board (IRB# 11-02-060E).

Note that full information on the approval of the study protocol must also be provided in the manuscript.

Field-specific reporting

Please select the one below that is the best fit for your research. If you are not sure, read the appropriate sections before making your selection.

☒ Life sciences ☐ Behavioural & social sciences ☐ Ecological, evolutionary & environmental sciences

For a reference copy of the document with all sections, see nature.com/documents/nr-reporting-summary-flat.pdf

Life sciences study design

All studies must disclose on these points even when the disclosure is negative.

Sample size	Sample sizes were determined based on the results of preliminary experiments or similar experiments previously published by the authors (John et al, Nat Comms, 2022; Ren et al, JCI, 2022; Wei et al, Sci Immunol, 2021). The sample sizes have sufficient statistical power to detect a treatment effect (p values < 0.05).
Data exclusions	One animal was excluded from Fig. 4j due to being statistical outliers as determined by Grubb's outlier test. For flow cytometric analyses, samples with very sparse cell populations, very poor cell viability, or poor quality sample preparation were excluded. All data exclusion criteria were pre-determined.
Replication	In vivo experiments were repeated twice after preliminary experiments with consistent data. In vitro experiments were successfully repeated three or more times, representative data was shown within the article and supplementary Information.
Randomization	For all tumor experiments, age-matched and sex-matched animals were randomized into control or treated groups after leukemia engraftment, prior to the start of monoclonal antibody treatment.
Blinding	Investigators were not blinded to group allocation during data collection or analysis, as investigators had to be aware of the groups during separation and treatment steps. Blinding was not needed for data analysis since identical or comparable analysis criteria were applied to all groups.

Reporting for specific materials, systems and methods

We require information from authors about some types of materials, experimental systems and methods used in many studies. Here, indicate whether each material, system or method listed is relevant to your study. If you are not sure if a list item applies to your research, read the appropriate section before selecting a response.

Materials & experimental systems

n/a	Involved in the study
☐	☒ Antibodies
☐	☒ Eukaryotic cell lines
☒	☐ Palaeontology and archaeology
☐	☒ Animals and other organisms
☒	☐ Clinical data
☒	☐ Dual use research of concern
☒	☐ Plants

Methods

n/a	Involved in the study
☒	☐ ChIP-seq
☐	☒ Flow cytometry
☒	☐ MRI-based neuroimaging

Antibodies

Antibodies used	All the primary and secondary antibodies (with supplier name, catalog number, clone name, lot number and dilution, as applicable), are listed in the Key resource table.
Validation	In this manuscript, the home-made anti-TMIGD2 mAbs, 17C7 and 20F2, have been validated in Supplementary Fig. 7b and c. The home-made anti-HHLA2 mAbs, A3H11 and B5B5, have been validated in our previous paper (Wei et al, Sci Immunol, 2021). The antibodies used for flow cytometry in this manuscript are commonly used, commercially available clones. In particular, the primary antibody vendors used in this project (Biolegend, BD, and R&D) carry out extensive validation processes for flow cytometry antibodies. These are selected from high-affinity clones, tested for binding across multiple assays, and/or specific binding confirmed by gene knockout of the target protein. The catalog#, clone name, and company for all antibodies are listed in the Key resource table. In this paper, expression of key markers including CD34, CD38, TMIGD2, CREB, and phospho-CREB was compared against isotype staining or fluorescence minus-one controls, and no non-specific staining was observed.

Eukaryotic cell lines

Policy information about cell lines and Sex and Gender in Research

Cell line source(s)	See Key Resources Table
Authentication	Cell lines were authenticated by the suppliers and further confirmed in the lab by FACS.
Mycoplasma contamination	The cell lines were routinely tested for mycoplasma contamination and confirmed to be negative. Cell lines were treated with prophylactic Plasmocin to prevent contamination prior to create long-term storage stocks.
Commonly misidentified lines (See ICLAC register)	No commonly misidentified lines were used.

Animals and other research organisms

Policy information about studies involving animals; ARRIVE guidelines recommended for reporting animal research, and Sex and Gender in Research

Laboratory animals	NOD/SCID IL2Rgammanull (NSG) and BALB/c female mice, 6-8 weeks old, were purchased from Jackson Laboratory and Charles River, respectively. The animals were housed in a specific pathogen free facility, in 12 hour light/12 hour dark cycles with temperatures maintained between 65F to 75F, and humidity maintained 40-60%.
Wild animals	Wild animals were not used.
Reporting on sex	For anti-TMIGD2 mAbs treatment of newly-established AML PDX models, sublethally irradiated (200 cGy) female NSG mice were transplanted with CD34+ primary AML cells followed by in vivo treatment with isotype control versus anti-TMIGD2 mAbs (200ug, i.p. every 3 days).
Field-collected samples	No field collected samples were used in the study.
Ethics oversight	Mice were bred and maintained in individual ventilated cages and fed with autoclaved food and water at the animal facility at Albert Einstein College of Medicine. All mouse studies were performed in compliance with approved protocols from the Institutional Animal Care and Use Committee at Albert Einstein College of Medicine

Note that full information on the approval of the study protocol must also be provided in the manuscript.

Flow Cytometry

Plots

Confirm that:

☒ The axis labels state the marker and fluorochrome used (e.g. CD4-FITC).

☒ The axis scales are clearly visible. Include numbers along axes only for bottom left plot of group (a 'group' is an analysis of identical markers).

☒ All plots are contour plots with outliers or pseudocolor plots.

☒ A numerical value for number of cells or percentage (with statistics) is provided.

Methodology

Sample preparation	Leukemia blasts and mononuclear cells were isolated using Lymphoprep (STEMCELL Technologies) density gradient separation and CD34+ cells were enriched using the CD34 Microbead kit (130-046-702, Miltenyi Biotec). Cells collected from spleen and bone marrow of NSG mice were lysed with 1X red blood cell lysis buffer before staining.
Instrument	Stained samples were analyzed on the BD LSR-II or BD FACS Calibur.
Software	BD FACS Diva 8.0 was used for acquisition, Flowjo 10.8 was used for data analysis.
Cell population abundance	FMO controls were used to determine appropriate positively-stained populations. In general, a stopping gate of 10,000 to 50,000 was used on total live CD45+ or CD33+ cells. Final gated populations generally ranged in 1000 to 10,000 cells, and samples with less than 50 events in the final gated population were excluded.
Gating strategy	Fig. 1a, 5f, 7f and Supplementary Fig. 1e, 1g, 2, 3h, 4i, 4j, 6i, 6j, 7e, 7g and 7l for gating strategies used in immunophenotyping.

☒ Tick this box to confirm that a figure exemplifying the gating strategy is provided in the Supplementary Information.
